# Estrogens and the Schrödinger’s Cat in the Ovarian Tumor Microenvironment

**DOI:** 10.3390/cancers13195011

**Published:** 2021-10-06

**Authors:** Marija Gjorgoska, Tea Lanišnik Rižner

**Affiliations:** Institute of Biochemistry and Molecular Genetics, Faculty of Medicine, University of Ljubljana, 1000 Ljubljana, Slovenia; marija.gjorgoska@mf.uni-lj.si

**Keywords:** ovarian cancer, estrogens, immunosuppressive microenvironment, plasticity

## Abstract

**Simple Summary:**

Ovarian cancer is a complex pathology for which we require effective screening and therapeutical strategies. Apart from the cancer cell portion, there exist plastic immune and non-immune cell populations, jointly constituting the context-adaptive tumor microenvironment, which is pivotal in tumorigenesis. Estrogens might be synthesized in the ovarian tumor tissue and actively contribute to the shaping of an immunosuppressive microenvironment. Current immune therapies have limited effectiveness as a multitude of factors influence the outcome. A thorough understanding of the ovarian cancer biology is crucial in the efforts to reestablish homeostasis.

**Abstract:**

Ovarian cancer is a heterogeneous disease affecting the aging ovary, in concert with a complex network of cells and signals, together representing the ovarian tumor microenvironment. As in the “Schrödinger’s cat” thought experiment, the context-dependent constituents of the—by the time of diagnosis—well-established tumor microenvironment may display a tumor-protective and -destructive role. Systemic and locally synthesized estrogens contribute to the formation of a pro-tumoral microenvironment that enables the sustained tumor growth, invasion and metastasis. Here we focus on the estrogen biosynthetic and metabolic pathways in ovarian cancer and elaborate their actions on phenotypically plastic, estrogen-responsive, aging immune cells of the tumor microenvironment, altogether highlighting the multicomponent-connectedness and complexity of cancer, and contributing to a broader understanding of the ovarian cancer biology.

## 1. Introduction

Ovarian cancer (OC) is a group of diseases affecting the aging ovary. The disease is usually detected at an already advanced stage due to its non-specific symptoms and deficiency of effective screening strategies. In 2020, the age standardized incidence and mortality rate of the disease worldwide were 6.6 and 4.2 per 100,000 population, respectively, whereas the 5-year prevalence was 21.3 per 100,000 population [1]. The median age at diagnosis worldwide is 61 [2]. Measurements of serum concentration of CA-125, a surface epithelial mucinous glycoprotein, and abdominal and transvaginal ultrasound are the standard investigations when OC is suspected [3]. The current gold standard of treatment encompasses cytoreductive surgery, including total hysterectomy, bilateral salpingo-oophorectomy, tumor debulking and omentectomy, followed by first-line chemotherapy, that is usually a combination of a DNA-damaging platinum compound (cisplatin, carboplatin) and a cell-cycle blocking compound taxane (paclitaxel, docetaxel) [4]. Response to the treatment is monitored radiologically, and by measurements of serum CA-125 levels. Even though the initial response to standard chemotherapy is over 80%, a relapse with a median progression free survival of 18 months is frequent, in which case patients receive a second-line chemotherapy [5]. New treatment options for OC patients with recurrent disease include the poly ADP-ribose polymerase inhibitors (PARPi), currently in phase 3 clinical trials [6], as well as cell-cycle modulators for refractory OC, currently in phase 2 clinical trial [7,8]. Clinical benefit has also been observed in patients subjected to a secondary cytoreductive surgery [9,10].

Contrary to the well-established link between estrogens and the pathogenesis of breast and endometrial cancer, the role of these sex steroid hormones in OC onset and development is not clear. Ovarian tumor tissue is responsive to estrogens [11], and estrogen exposure represents a risk factor for the disease [12]. Moreover, systemic levels of estrogens and their metabolites are altogether higher in OC patients [13], and several folds greater in the ovarian tumor tissue, compared to the systemic circulation [14], together indicative of a local estrogen synthesis in the tumor itself. In addition, the pool of estrogens available to the ovarian tumor might be additionally modulated by the changing microbiome [15,16]. Despite these indications of association between OC and estrogens, therapeutic approaches targeting estrogen receptors or inhibitors of the estrogen biosynthesis pathway are modestly beneficial for a certain group of OC patients [17]. The heterogeneity of the disease, as well as its diagnosis usually at an already advanced stage, when an immunosuppressive microenvironment has been well established beforehand, might be an explanation for the limited effectiveness of these therapeutic approaches, or other single-agent therapeutic approaches. 

Here we review the biosynthetic and metabolic pathways of estrogens and elaborate their contributing role in establishing a tumor-promoting microenvironment, the latter being referred to in the title as the Schrödinger’s cat. Briefly, the Schrödinger’s cat is a thought experiment in quantum physics describing a paradox, where a cat in a box is simultaneously alive and dead, its final state depending on the state of a radioactive atom. Drawing the correlation from quantum physics to the tumor microenvironment, we observe a similar superposition, with various cell populations existing in a phenotypic continuum; the two extremes: anti- and pro-tumorigenic phenotypes corresponding to the alive and dead state of the cat. Contrary to the cat, whose fate is decided by a single random subatomic event that may or may not occur, the fate of the microenvironment is influenced by a plethora of factors, and estrogens are one of them.

## 2. Epithelial Ovarian Cancer Classification

Surface epithelial ovarian neoplasms represent 65% of ovarian neoplasms; the latter are based on atypia, or structural abnormality, classified into benign (cystadenoma), intermediate (tumor of low malignant potential/of borderline malignancy), and malignant (cancer). Surface epithelial OC accounts for 90% of malignant ovarian neoplasms, and based on the tumor cell type is classified into four major subtypes, namely serous, mucinous, endometrioid and clear cell epithelial OC [18].

Serous epithelial OC accounts for the majority of epithelial OC cases [19]. Two histotypes and genetically distinct forms are distinguished, namely high-grade and low-grade serous OC (HGSOC and LGSOC). The HGSOC accounts for more than 90% of serous epithelial OC and 70% of deaths [20], and it is characterized by severe nuclear atypia, high nuclear-to-cytoplasmic ratio, abundant mitosis and chromosomal instability [4]. Molecularly, HGSOC is characterized by pathognomonic mutations in the tumor-suppressor *TP53* gene, and mutations in *BRCA* genes [21]. The HGSOC derives from the fallopian tube epithelium (FTE) and the ovarian surface epithelium (OSE); the origin confers distinct transcriptional and proteomic profile as well as different response to chemotherapy [22,23,24]. The formation of HGSOC from FTE involves early *TP53* mutations in the normal FTE, leading to the formation of serous tubal intra-epithelial carcinomas (STICs), which will eventually seed in the ovaries in a matter of years, resulting with an already advanced HGSOC, capable to metastasize rapidly thereafter [25]. When the same genetic lesions develop in the OSE, an OSE-derived HGSOC forms [23].

The LGSOC accounts for approximately 5% of serous epithelial OC, and it is characterized by mild to moderate nuclear atypia, lower nuclear-to-cytoplasmic ratio, relative chromosomal stability, and less aggressive behavior, in comparison to HGSOC [26]. Molecularly, LGSOC is characterized with mutations in *KRAS*, *BRAF*, *ERBB2*, and *NRAS*, genes involved in the mitogen activated protein kinase (MAPK) pathway [27]. The LGSOC is considered to originate from the FTE that upon morphogenetic transformation induces ovarian epithelial inclusions, which then evolve to benign cystadenoma, subsequently to borderline ovarian neoplasm and finally to LGSOC [28,29].

Mucinous epithelial OC accounts for 3–5% of all epithelial OC [30], and it is characterized by atypical endocervical-type and/or intestinal-type cells filled with mucin [18], and by prevalent *KRAS* mutations [31]. The mucinous OC was considered to develop from metastatic tumors mainly from the lower gastrointestinal tract [32]. A non-metastatic origin of mucinous OC has recently been suggested, arising from mucinous benign cystadenoma that has been initiated with a mutation in *KRAS* or *CDKN2A* gene, which by accumulating additional genomic alterations then progresses to mucinous borderline neoplasm, localized low-grade mucinous OC, and finally to high-grade invasive mucinous OC [33].

Endometrioid and clear-cell epithelial OC are epidemiologically and molecularly associated with endometriosis [34], and account for 10% and 6% of all epithelial OC, respectively [19]. Molecularly, they are characterized by mutations in *ARID1A*, *PIK3CA* and *PTEN* genes, mutations coexisting in endometriosis and endometrial cancer [35,36]. The high-grade endometrioid OC, on the other hand, more closely resembles the HGSOC, harboring mutations in *TP53*, as well as copy number variations [37]. The origin of endometrioid and clear-cell OC is not yet clear; both the formation from neoplastic endometrial [38] and FTE cells [39] have been suggested.

Numerous risk factors of developing OC are known; some of them are increasing age, germline mutations in the homologous recombination DNA repair pathway, particularly in *BRCA1/BRCA2* genes and mutations in mismatch repair genes [40,41]. Oral contraceptive use, parity, tubal ligation, uni- and bilateral salpingectomy, uni- and bilateral oophorectomy, and hysterectomy on the other hand, reduce the risk of developing OC [4].

## 3. OC and Estrogens

OC affects a once-main estrogen-producing organ, and multiple lines of evidence indicate a connection between OC tumorigenesis and estrogens. For instance, exposure to hormone replacement therapy (HRT), consisting of estrogen-only and estrogen-progestin combination, is regarded as a risk factor for developing OC, especially the serous and endometrioid subtype [12,42]. Moreover, OC patients have higher serum levels of estrogens and methylated estrogen metabolites, than a control group [13], and several folds greater estrogen levels in the tumor itself than in serum [14]. Furthermore, estrogen levels have been positively associated with the development of non-serous OC [43], particularly endometrioid OC [44]. In continuation, we discuss how estrogen signaling affects the ovarian tumor tissue through various receptor isoforms, and elaborate the estrogen biosynthetic and metabolic pathways in both, physiological and pathological state. 

### 3.1. Estrogen Signaling in OC

Estrogen action is mediated by estrogen receptors (ERs). The ligand-activated nuclear and cytoplasmic ERs α and β, the former having four, the latter five known isoforms, exert genomic actions by binding to estrogen-response elements found in the promoter region of estrogen-regulated genes [45]. The non-genomic signaling on the other hand, mainly involves a membrane-bound G-protein coupled ER (GPER), which mediates rapid kinase activation and transcriptional events upon ligand binding [46].

The normal ovary expresses predominantly ERβ; however, the ERα/ERβ ratio seems to change in favor to ERα in the progression from normal ovary to primary OC to metastatic disease [47,48]. The proportion of ERα-positive tumors is high in HGSOC, LGSOC, and endometrioid OC, 81%, 88% and 77%, respectively, and modest in mucinous and clear cell OC, 21% and 20%, respectively [11]. In OC, estrogen signaling via ERα has been associated with growth stimulation, cell migration, and epithelial–mesenchymal transition, and thus with poorer survival [49]. Estrogen signaling via the ERβ, on the other hand, is more variable; the ERβ2 and ERβ5 isoforms have been associated with proliferative, pro-migratory and invasive activities, whereas the ERβ1 isoform with better overall survival [50]. The role of ERα/ERβ heterodimers in OC remains poorly understood; some evidence indicates that ERβ opposes the action of ERα [51]. Likewise, the role of GPER in OC remains elusive, as both a protective role [52], and a correlation with poor survival [53] have been reported.

### 3.2. Estrogen Synthesis in a Physiological State 

**Figure 1:**
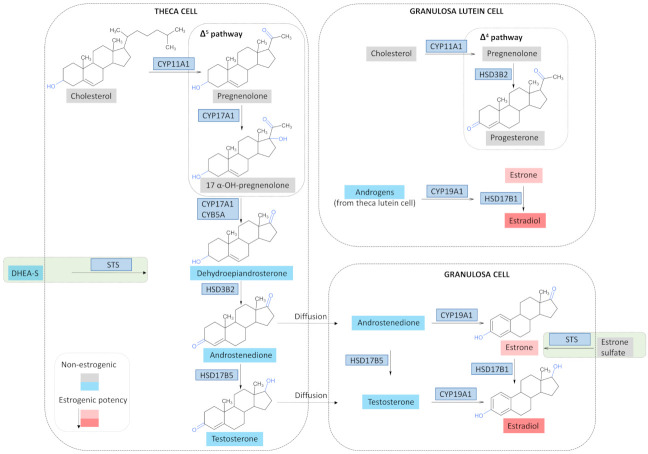
Ovarian steroidogenesis. During a female reproductive age, the thecal and granulosa cells surrounding the developing ovarian follicle and the corpus luteum synthesize progestagens, androgens and estrogens; the Δ^5^ pathway converts pregnenolone to 17-α-pregnenolone, which subsequently gives rise to androgens and estrogens, and is the preferred pathway in thecal cells. The Δ^4^ pathway converts pregnenolone to progesterone, and is the main pathway in the granulosa lutein cells of the corpus luteum; however, estrogen synthesis also occurs. In OC tissue, local estrogen synthesis might take place from circulating androgen (dehydroepiandrosterone sulfate (DHEA-S), dehydroepiandrosterone (DHEA), androstenedione) (left green box) and estrogen precursors (estrone sulfate, estrone) (right green box) via the aromatase and sulfatase pathway, respectively. CYB5A, cytochrome b_5_ type A; CYP11A1, cytochrome P450 cholesterol side chain cleavage; CYP17A1, cytochrome P450 17A1; CYP19A1, cytochrome P450 19A1 (aromatase); HSD3B2, 3β-hydroxysteroid dehydrogenase/Δ^4/5^ isomerase type 2; HSD17B1, 17β-hydroxysteroid dehydrogenase type 1; HSD17B5, 17β-hydroxysteroid dehydrogenase type 5; OH, hydroxy; STS, sulfatase. Parts of the figure were drawn using ACD/ChemSketch, version 2021.1.1, Advanced Chemistry Development, Inc., Toronto, On, Canada, www.acdlabs.com, accessed on 5 May 2021.

During the female reproductive period, the ovaries synthesize three classes of steroid hormones under the control of the hypothalamic-pituitary-ovarian axis, namely progestagens, androgens and estrogens, each having a profound effect on the female reproductive organs. Ovarian steroidogenesis takes place in the inner layer of thecal cells and granulosa cells that surround the ovarian follicles (Figure 1). The synthesis begins with cholesterol, mainly provided by plasma low-density lipoproteins (LDL), although de novo synthesis from acetate happens as well. Cholesterol is then transferred from the cytoplasm to the outer mitochondrial membrane by an unknown mechanism, and subsequently to the inner mitochondrial membrane by the mediation of the steroidogenic acute regulatory (StAR) protein. The subsequent conversion of cholesterol to pregnenolone is catalyzed by the mitochondrial cholesterol side-cleavage enzyme (CYP11A1). This rate-limiting and hormonally regulated step involves three sequential chemical reactions: 20α-hydroxylation, 22-hydroxylation, and scission of the C20-C22 bond [54]. Once cholesterol has been converted to pregnenolone, androgen or progesterone formation takes place. The former is the dominant steroidogenic pathway during the follicular phase; the latter predominates during the luteal phase. 

In the follicular phase, pregnenolone conversion to dehydroepiandrosterone (DHEA) happens in the thecal cells. This is mediated by CYP17A1 which has a 17α-hydroxylase and a 17,20-lyase activity [55]. First, a 17α hydroxylation leading to 17-α-OH-pregnenolone happens, followed by a cleavage of the C17-C20 bond. The enzyme 3β-hydroxysteroid dehydrogenase type 2 (HSD3B2) then converts the resulting DHEA to androstenedione. The reaction first involves a conversion of the hydroxyl group at C3 to a keto group, and a subsequent isomerization of the double bond from the B ring (Δ5 steroid) to the A ring (Δ4 steroid) [56]. Androstenedione is then converted to testosterone by the action of 17β-HSD type 5 (HSD17B5), a member of the aldo-keto reductase family, better known as AKR1C3. The thecal androgens then diffuse to the granulosa cells; however, a portion also enters the systemic circulation. The granulosa cells express exclusively CYP19A1, an enzyme responsible for the conversion of androstenedione and testosterone from the thecal cells to estrogens. Type I 17β-HSD (HSD17B1) catalyzes the last step of estradiol synthesis. In the luteal phase, granulosa and theca cells differentiate under the influence of LH into granulosa and theca lutein cells and thus form the corpus luteum. Thecal lutein cells continue to provide androgens to the granulosa lutein cells, which now express StAR, CYP11A1, and HSD3B2, but not CYP17A1. This permits the granulosa lutein cells to produce large amounts of progesterone, while still converting the C-19 steroids from the theca to estrogens. 

With the onset of menopause, the production of steroid hormones by the ovaries reduces significantly; however, it does not completely diminish, as androgen synthesis still occurs [57]. Nonetheless, the extra-gonadal sites, such as the adrenal glands, liver, brain, bone, vascular endothelium, adipose tissue are considered as primary source of androgens and estrogens in postmenopausal women [58].

### 3.3. Estrogen Synthesis in OC

Epithelial ovarian tumor tissue and the ovarian vein draining from the tumorous ovary contain several folds greater estrogen levels than serum [14,59], and intriguingly, serum estradiol concentration seems to decrease after tumor surgery [60,61]. These data are indicative that the ovarian tumor might as well be an estrogen source fueling tumor growth. Indeed, markers of sex-steroid differentiation, as well as steroidogenesis-related enzymes express in the stroma adjacent to ovarian tumors, and this is especially prominent in the endometriosis-associated OC and mucinous OC [59,62]. In addition, sulfated androgen and estrogen precursors, present in significant quantities in postmenopausal women, can give rise to estrogens via two biochemically distinct pathways, namely, the aromatase and sulfatase pathway (Figure 1). The aromatase pathway involves estrogen synthesis from the androgen precursors DHEA-S, DHEA or androstenedione and testosterone, by the action of the sulfatase (STS), oxidative 3βHSDs, reductive 17βHSDs and aromatase CYP19A1 enzymes, consecutively. The sulfatase pathway involves estrogen synthesis from the estrogen precursor estrone-sulfate (E1-S), by the actions of STS and the reductive 17βHSD [63]. The enzymes of both pathways express in ovarian tumor tissues [64].

### 3.4. Estrogen Metabolism and the Microbiome Change in OC

**Figure 2:**
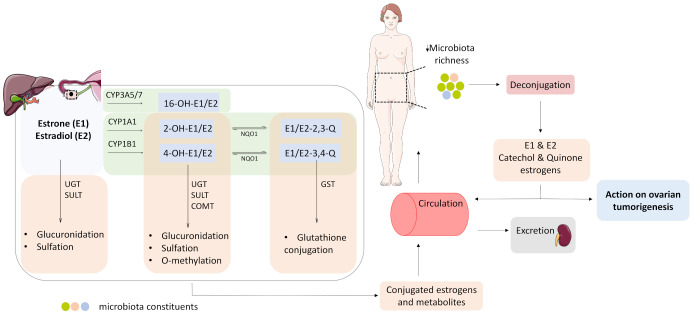
Estrogen metabolism in the liver and in the ovarian tumor tissue. Oxidation (light green boxes) is catalyzed by cytochrome P450 enzymes, and gives rise to catechol-estrogens: 2- and 4- OH estrone/estradiol, and 16-OH-estrone/estradiol (2-OH-E1/E2 and 4-OH-E1/E2, 16-OH-E1/E2). 2- and 4- catechol-estrogens are further oxidized to DNA-damaging quinones (E1/E2-2,3-Q and E1/E2-3,4-Q). Conjugation (light orange boxes) involves glucuronidation, catalyzed by UDP glucuronosyl transferases (UGT2B7), sulfation, catalyzed by sulfotransferases (SULT1A1, SULT1E1, SULT2B1), methylation, catalyzed by catechol-O-methyl transferase (COMT), and glutathione conjugation, catalyzed by glutathione S-transferases (GSTP1). The conjugated estrogens and their metabolites enter the systemic circulation and are eventually excreted from the body through urine and feces. The microbiome, more precisely the estrobolome, actively modulates the estrogen levels in the body. The microbiome change observed in OC patients, might contribute to the pool of estrogens available to the estrogen-responsive ovarian tumor by de-conjugating already conjugated estrogens and estrogen metabolites, hence to ovarian tumorigenesis. COMT, catechol-ortho-methyl transferase; CYP1A1, cytochrome P450 1A1; CYP1B1, cytochrome P450 1B1; CYP3A5, cytochrome P450 3A5; CYP3A7, cytochrome P450 3A7; GST, glutathione-s-transferase; NQO1, NAD(P)H quinone oxidoreductase 1; SULT, sulfotransferase; UGT, uridine 5’ diphospho-glucuronosyl transferase. Parts of the figure were drawn by using pictures from Servier Medical Art. Servier Medical Art by Servier is licensed under a Creative Commons Attribution 3.0 Unported License (https://creativecommons.org/licenses/by/3.0/) (accessed on 7 May 2021).

The estrogen metabolism occurs predominantly in the liver, and involves an oxidation and a conjugation phase (Figure 2). The cytochrome P450 enzymes catalyze the oxidation phase, resulting with the formation of hydroxylated estrogens, namely 2-, 4-, and 16-hydroxy estrogens; the former two eventually give rise to DNA-damaging 2- and 4-estrogen quinones. The conjugation phase involves glucuronidation mediated by UDP-glucuronosyltransferases (UGTs), sulfation by sulfotransferases (SULT), methylation by catechol-o-methyl transferase (COMT) and glutathione conjugation by glutathione S-transferases (GST), resulting in soluble estrogen metabolites that are eventually excreted from the body [65].

Interestingly, the enzymes involved in the first and second phase of the estrogen metabolism express in OC tissues as well, suggesting that ovarian tumor tissue might metabolize estrogens locally (Figure 2). Furthermore, the CYP450 enzymes CYP1A1 and CYP1B1 catalyzing the formation of 2- and 4-catechol estrogens, respectively, are present at a higher level in cancerous compared to normal ovaries [66]. In addition, the expression of NQO1, a NAD(P)H quinone oxidoreductase that catalyzes the reduction of estrogen quinones back to catechols, correlates with a higher histological grade, advanced clinical stage and lower overall survival rates in OC patients [67]. Ovarian tumors express conjugation phase enzymes as well; moreover, the GST class-pi (GSTP1) enzyme has been reported as one of the most abundantly expressed genes in ovarian tumors [68], whereas the sulfotransferase SULT1E1 as an independent positive prognostic factor in HGSOC [69].

Estrogen levels available to the estrogen-responsive ovarian tumor tissue can be modulated by the human microbiome, i.e., a collection of genomes from all microorganisms that are part of the human body, more specifically, by the estrobolome, i.e., a group of microbial genes capable of producing estrogen-metabolizing enzymes [15]. The de-conjugating enzymes glucuronidases (GUS) and sulfatases are the most well studied part of the estrobolome, and can effectively reverse the excretion of conjugated estrogens and estrogen metabolites and repurpose them to other sites of the body [70]. GUS activity has been noted in the major phyla of the mammalian gut microbiota Bacteroidetes, Firmicutes, Verrucomicrobia and Proteobacteria [71], whereas in the case of the microbial sulfatases, a great structural and functional diversity and active sites differing from those of the human sulfatases have been observed [72].

The microbiome changes during OC pathogenesis, which is not surprising, given the plasticity of the former per se. Changes in the composition of the microbial community in the gut [73], vagina and cervix [74], and peritoneum [75], as well as in the ovarian tumor tissue itself [16] have been observed in OC patients. Moreover, the gut microbiota changes post-operatively, and this it is characterized by an increase in the abundance of Proteobacteria and a decrease in abundance of Bacteroidetes and Firmicutes, whereas exactly the opposite change takes place after exposure to chemotherapy [73]. The change in the cervico-vaginal microbiota involves a reduction of *lactobacilli* species [74], whereas in the ovarian tumor tissue, an overall reduction in microbiota biodiversity and richness takes place [16]. The diversity of the microbiome affects estrogen metabolism and correlates positively with conjugation and urinary excretion of estrogen metabolites in healthy postmenopausal women [15,76]. The distinct changes taking place in OC might contribute to a shift towards estrogen de-conjugation (Figure 2).

## 4. The Emergence of a Tumor-Permissive Microenvironment and the Role of Estrogens

Up until now, we have discussed the biochemical pathways of estrogen synthesis, metabolism and signaling in the estrogen-responsive, enzymatically equipped ovarian tumor. The following question might arise for the reader: if pro-tumoral estrogens are synthesized and act on the ovarian tumor, why then do inhibitors of estrogen biosynthesis or estrogen receptor antagonists have but little effect on disease progression? An answer might be found by putting estrogen signaling into a broader context, i.e., in the realm of the growingly complex tumoral microenvironment.

The normal ovarian tissue is composed of distinct cell types, such as oocytes, granulosa, stromal, immune, endothelial, perivascular cells [77], and undergoes changes over the course of aging [78]. This tissue microenvironment acts as a barrier to tumorigenesis, by exerting elimination pressure to emerging tumor cells [79,80]. The cancer-immunity cycle begins with the uptake of neo-antigens released by tumor cells by professional antigen presenting cells (APCs). Next, the APCs present the processed neo-antigens to T cells in the tumor-draining lymph nodes, resulting in the priming and activation of effector T cell responses; for this, additional robust signals specifying immunity to the effector T cells are indispensable [81,82,83]. Finally, the activated T cells traffic to and infiltrate the tumor bed, specifically recognize and kill target tumor cells; the latter then release additional neo-antigens, thus induce subsequent revolution of the cycle, and eventually constrain the malignant phenotype [84].

**Figure 3:**
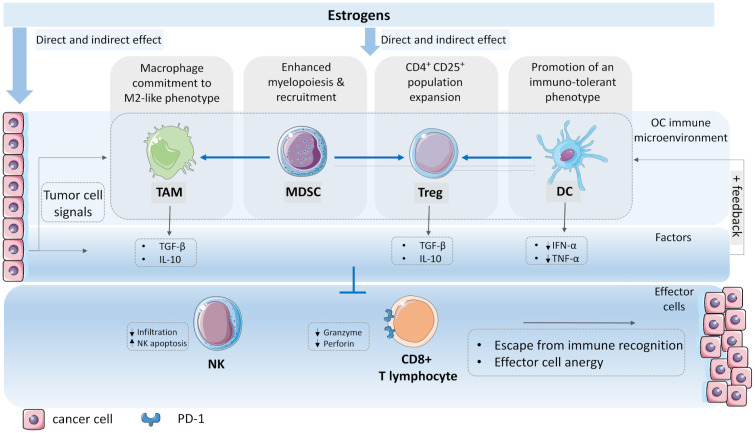
The immunosuppressive ovarian tumor microenvironment and the contribution of estrogens to its formation. Local and systemic estrogens reaching the ovarian tumor tissue affect various cells of the growingly immunosuppressive microenvironment. Immune cells are responsive to estrogens through receptors. Estrogens contribute to M2-like activation of macrophages and their accumulation in the tumor tissue. Furthermore, estrogens might enhance pathologic myelopoiesis, recruitment of immature myeloid cells (IMCs) to the tumor tissue and their conversion to myeloid derived suppressor cells (MDSCs). Moreover, estrogens stimulate the expansion of regulatory T (T_reg_) cells, and might promote tolerogenic phenotype in dendritic cells (DCs). In addition, these immune cells of the microenvironment influence the activity of each other, for example, MDSCs might commit to M2-like tumor associated macrophages (TAMs) and T_regs_, which in turn inhibit DCs antigen-presenting actions. A plethora of soluble factors, such as TGF-β, IL-10 released in the microenvironment further stimulate the formation of an immunosuppressive microenvironment that inhibits effector CD8^+^ cells and NK cells. Consequently, CD8^+^ T lymphocytes present as less mature, produce less granzyme and perforine, and overexpress PD-1, which drives them to exhaustion. The inhospitable microenvironment restricts the NKs infiltration and induces their apoptosis, altogether leading to tumor escape from immune destruction. Parts of the figure were drawn by using pictures from Servier Medical Art. Servier Medical Art by Servier is licensed under a Creative Commons Attribution 3.0 Unported License (https://creativecommons.org/licenses/by/3.0/) (accessed on 7 May 2021).

At a certain point, however, the microenvironment can change from being a tumor-suppressive to a tumor-promoting ecosystem that creates pro-tumorigenic conditions where cancer cells flourish [85]. Unfortunately, we do not yet know which cell type of the microenvironment starts to deflect first, or whether there is a master deflection capable of inducing a cascade of other deflections, ultimately leading to immune override. We know, however, that in the process, it is not necessarily that whole cell populations have to switch from anti- to pro-tumorous, but rather subpopulations within cell populations, e.g., fibroblasts, macrophages, neutrophiles, emerge, and then this variety of subpopulations with completely different functional programs may exert either pro- or anti-inflammatory actions, thus influencing the disease outcome [86,87]. In continuation, we explore the emergence of the ovarian tumor-permissive microenvironment, with a focus on its immune portion, and highlight the role of estrogens in the building of inhospitable conditions that ultimately exhaust the effector immune cells, thus granting the cancer cells an escape from immune recognition (Figure 3). 

### 4.1. Macrophage Commitment to Tumor-Associated Type

Macrophages are an immune cell population found in association with ovarian epithelial cells when the latter are transformed into metaplastic cells [88]. The macrophages might be tissue residents derived from the embryonic sac, or derived from peripheral reservoirs such as the bone marrow and spleen [89]. A hallmark of this immune population is its functional plasticity, i.e., alteration of the polarization state in response to different physiological conditions, with classically activated, pro-inflammatory M1 and alternatively activated, anti-inflammatory M2 macrophages being the two extremes of their phenotypic continuum [90,91,92].

In the ovarian tumor microenvironment, macrophages represent the main population of immune cells, and are regarded as tumor-associated macrophages (TAMs), which tend to display an M2-like phenotype [93]. Higher macrophage infiltration is observed in cancerous compared to benign lesions; thus, TAM infiltration represents a poor prognostic predictor of OC [94]. In addition, higher serum levels of the M2 marker CD163, and higher ratio of CD163^+^ cells to total macrophages [95] correlate with a higher tumor grade, worse progression-free survival, and early relapse of serous OC after first-line chemotherapy [96]. Conversely, higher resting macrophage (M0) population, as well as higher M1/M2 ratio are associated with a better overall survival in epithelial OC patients [97,98]. 

The mechanisms that initiate the macrophage switch from tumor-suppressing to tumor-promoting are multifaceted, and estrogens are a significant contributing factor (Figure 3). Macrophages express ERs and GPER, the effect of the former seems to be context dependent and to augment as well as to dampen innate immune signaling pathways in macrophages [99]. The ERα is regarded as the key mediator of the estrogen anti-inflammatory activity in these cells [88,100,101]. Moreover, ERα-positive ovarian tumors have higher intra-tumoral TAM density after exposure to exogenous estradiol [102]. Apart estrogens, a plethora of soluble factors present in the microenvironment, such as interleukins (IL)-4, -6, -10, -13, transforming growth factor β (TGF- β), colony-stimulated factor 1 (CSF-1), strongly favor the alternative macrophage state [103]. Environmental conditions such as hypoxia, platinum exposure, might promote the formation of M2-like TAMs as well [104].

Once formed, pro-tumoral TAMs affect the ovarian tumor microenvironment by the secretion of anti-inflammatory cytokines, such as IL-4, -5, -6, -10, as well as tumor necrosis factor α (TNF- α) and TGF-β [105], altogether impairing the activity of other immune cells in the tumor microenvironment (Figure 3). In addition, TAMs might contribute to chemoresistance, for instance by upregulating multidrug resistance genes in cancer cells [106].

### 4.2. Recruitment of Myeloid Derived Suppressor Cells

Myeloid-derived suppressor cells (MDSC) are an immature myeloid cell population characterized by an exceptional ability to suppress T cell responses [107]. In physiological conditions, immature myeloid cells (IMCs) from the bone marrow give rise to macrophages, dendritic cells and granulocytes [108]. In cancer, IMCs accumulate and expand to the tumor sites; however, the tumor microenvironment prevents their differentiation, and instead immunosuppressive MDSCs emerge. Two subsets of MDSC are distinguished, namely granulocytic and monocytic. Patients with epithelial OC have higher monocytic MDSCs count in the peripheral blood, compared to controls [109,110], and increased tumor-infiltrating MDSCs [111], which correlate with higher tumor aggressiveness and decreased survival [110,112].

Numerous activation factors within and beyond the tumor microenvironment might promote pathologic myelopoiesis, MDSCs mobilization and potentiation into the ovarian tumor microenvironment, and estrogens are among them (Figure 3). More specifically, estrogens have been shown to contribute to a deregulated myelopoiesis in OC via the JAK-STAT pathway [113]. Moreover, estrogens might drive MDSC mobilization and enhance their immunosuppressive activity in vivo, for example by promoting TNF-α secretion [114]. Indeed, a decreased MDSCs accumulation in liver metastasis, that is reversible by estradiol reconstitution, was observed in ovariectomized mice [115]. In addition, other factors from the tumor microenvironment, such as interferon γ (IFN-γ) and TGF-β, can promote MDSC expansion and inhibit their differentiation to maturity as well.

MDSCs in turn, affect the ovarian tumor microenvironment by disrupting the activity of other cells involved in the immune surveillance. For instance, MDSCs negatively affect the activity of dendritic cells, T cells, and natural killer cells, but promote de novo development of regulatory T cells in vivo [116]. Moreover, MDSCs can differentiate into TAMs [117], thus reinforcing the growing immune suppression (Figure 3).

### 4.3. Expansion of Regulatory T Cell Population

Regulatory T cells (T_reg_) comprise a minor population of thymus-derived CD4^+^ T cells that co-express the CD25 antigen and forkhead box P3 (FOXP3), and their main function is to control immune responses to autoantigens [118]. This cell population is part of the ovarian tumor microenvironment, and can be both, thymus-derived and locally induced. High levels of CD4^+^ CD25^+^ T cells have been identified in ovarian tumor masses and in malignant ascites of patients with untreated malignant epithelial OC [119]. Moreover, T_reg_ infiltration has been associated with advanced OC stage and reduced survival [120].

As in the case of TAMs and MDSCs, a multitude of immunosuppressive factors can drive T_reg_ population expansion and potentiate their immunosuppressive functions, and estrogens are among them (Figure 3). The latter promote T_reg_ population expansion, and this might be mediated by ERα [121,122]. Moreover, estrogens are capable of inducing naïve CD4^+^ CD25^−^ T cells and promoting their differentiation into T_regs_ in vitro [123]. Once formed, T_reg_ cells release factors, such as IL-10 and TGFβ, which directly inhibit effector cell function (Figure 3) [124,125].

### 4.4. The Dendritic Cell Immunotolerance

Dendritic cells (DCs) are specialized and robust APCs with key roles in the initiation and regulation of innate and adaptive immune responses [126]. Upon stimulation by danger-associated molecular patterns (DAMPs) from tumor cells, immature DCs migrate into lymph nodes, and gradually mature into three main groups, namely conventional (cDCs), monocyte-derived (mDCs), and plasmacytoid (pDCs) [127,128]. pDCs are the main subtype of DCs in the tumor sites of OC, and their infiltration in the OC microenvironment correlates negatively with prognosis, due to their low antigen presentation efficacy [129].

Estrogens affect DCs; the effect seems to be context- and dose-dependent [99,130]. For instance, estrogens can potentiate immune response by promoting DC differentiation [130], or dampen the immune response, by epigenetic alteration of gene expression programs governing DCs functions, or by promoting a tolerogenic DC phenotype [131]. Accumulating evidence suggests that DCs recruited to the tumor microenvironment often display a non-activated state, and by producing less IFN-α, TNF-α, and co-stimulating T_reg_ cells [132,133], promote immunosuppression (Figure 3). 

## 5. The Immune Override

The CD8^+^ T cells and the natural killer (NK) cells are the effector cells that have the capacity to eliminate neoplastic cells; however, all the actions described above seem to contribute to their inhibition. The effector CD8^+^ T cells are the major force of adaptive immunity, whose function is to activate, expand, and acquire subtype-specific effector functions upon antigen exposure, including the production of effector cytokines and granzyme/perforin mediated cytotoxicity [134,135]. NK cells, on the other hand, are effector lymphocytes of innate immunity, which can eliminate transformed cells without prior sensitization [136].

In OC, CD8^+^ T cell and NK cell infiltration correlate with an improved outcome [98,137]; unfortunately, these cell types are poorly represented, immature [138], or less potent [139], thus granting the cancer cells an immune escape. The inhospitality of the tumor microenvironment greatly contributes to this outcome (Figure 3). For instance, the soup of soluble factors in the microenvironment induces upregulation of the programmed death ligand 1 (PD-L1) on cancer and stromal cells; PD-L1 then binds to the matching PD-1 receptor expressed on CD8^+^ cells, ultimately leading to their exhaustion [140,141,142]. These factors contribute to the NKs’ depletion as well, by either lowering their attraction to the microenvironment or inducing in situ apoptosis [143], eventually granting the tumor an escape from immune destruction. Currently, several checkpoint inhibitors, such as anti-PD-1 and anti-PD-L1 inhibitors, are being tested as monotherapy and in combination with cytotoxic chemotherapy; unfortunately, only a modest benefit has been observed [144,145,146].

## 6. Conclusions

Here we discussed multiple aspects of OC pathogenesis and highlighted the role of estrogens. First, we covered the estrogen biosynthetic and metabolic pathways, which might take place in the ovarian tumor itself, and then we focused on the effect of estrogens on the ovarian tumor microenvironment. 

The correlation between the former and the latter poses questions, how and to which extent estrogens and the microenvironment influence each other. More specifically, does a modulation of the estrogen synthetic and metabolic pathways happen early in tumorigenesis to fine-tune estrogen levels, which in turn support the formation of a growingly complex tumor microenvironment, or does the microenvironment first become tumor-permissive, and is then steadily reinforced by a multitude of factors, including estrogens. More importantly, to what extent do these hormones influence the outcome of immune therapy? Can a lasting effect be achieved with immune therapy, when immune modulators, such as estrogens, are still present in the microenvironment? Is the microenvironment already too complex for an endocrine therapy to be applied? 

We believe these questions to be important now, when many immune checkpoint inhibitors are being developed and tested, yet the biology of OC is not thoroughly understood.

## 7. Future Directions

The main problem in OC management remains the lack of effective screening strategies; therefore, the search for reliable biomarkers is of utmost importance in tackling the disease. One approach might be to focus on the post-operative period and decipher patterns specific for recurring OC, which might also mirror changes that take place in a pre-cancerous state. Although the approach certainly has its limitations, such as the patient’s exposure to chemotherapy, it might provide a much-needed glimpse into the early changes in OC tumorigenesis.

Regarding therapeutic strategies, more integrated approaches, such as re-educating the tumor microenvironment to exert immune destruction and tumor elimination are most promising, although they might still be a long way away, due to our incomplete understanding of the biology of OC. Again, the post-operative period might be a great window of opportunity for establishing homeostasis and a competitive immune defense to a possible tumor reemergence, especially since a great part of the immunosuppressive source has been removed with the tumor itself, and the chemical gradients driving a shift to ineffective defense are reduced or absent. The post-operative period is also a period when great care should be taken with regard to the agents to which the patent is exposed, especially due to the dualistic nature of the microenvironment, which is why the tumor developed in the first place.
